# Pressure Pain Thresholds Increase after Preconditioning 1 Hz Repetitive Transcranial Magnetic Stimulation with Transcranial Direct Current Stimulation

**DOI:** 10.1371/journal.pone.0092540

**Published:** 2014-03-21

**Authors:** Tonya M. Moloney, Alice G. Witney

**Affiliations:** Department of Physiology, Trinity College Institute of Neuroscience and Trinity Centre for BioEngineering, Trinity College, Dublin, Ireland; University Medical Center Goettingen, Germany

## Abstract

**Background:**

The primary motor cortex (M1) is an effective target of non-invasive cortical stimulation (NICS) for pain threshold modulation. It has been suggested that the initial level of cortical excitability of M1 plays a key role in the plastic effects of NICS.

**Objective:**

Here we investigate whether transcranial direct current stimulation (tDCS) primed 1 Hz repetitive transcranial magnetic stimulation (rTMS) modulates experimental pressure pain thresholds and if this is related to observed alterations in cortical excitability.

**Method:**

15 healthy, male participants received 10 min 1 mA anodal, cathodal and sham tDCS to the left M1 before 15 min 1 Hz rTMS in separate sessions over a period of 3 weeks. Motor cortical excitability was recorded at baseline, post-tDCS priming and post-rTMS through recording motor evoked potentials (MEPs) from right FDI muscle. Pressure pain thresholds were determined by quantitative sensory testing (QST) through a computerized algometer, on the palmar thenar of the right hand pre- and post-stimulation.

**Results:**

Cathodal tDCS-primed 1 Hz-rTMS was found to reverse the expected suppressive effect of 1 Hz rTMS on cortical excitability; leading to an overall increase in activity (*p*<0.001) with a parallel increase in pressure pain thresholds (*p*<0.01). In contrast, anodal tDCS-primed 1 Hz-rTMS resulted in a corresponding decrease in cortical excitability (*p*<0.05), with no significant effect on pressure pain.

**Conclusion:**

This study demonstrates that priming the M1 before stimulation of 1 Hz-rTMS modulates experimental pressure pain thresholds in a safe and controlled manner, producing a form of analgesia.

## Introduction

Pain perception and modulation have become crucial topics of exploration within the scientific community due to increasing numbers seeking treatment for pain and the current inadequacy of available therapies. Chronic, persistent pain is a complex condition that has been identified as one of the most disabling and costly afflictions in North America, Europe, and Australia with a lifetime prevalence of up to 55.2% in the adult population [1].

In particular, musculoskeletal pain is a cause of long term pain and disability associated with common disorders such as rheumatoid arthritis, osteoarthritis and fibromyalgia. Algometry is used for the assessment of pressure pain thresholds in these patients with chronic musculoskeletal pain. Measurement of these pressure pain thresholds and assessment of the defined 18 tender points forms part of the American College of Rheumatology (ACR) guidelines for the diagnosis of fibromyalgia [2]. Testing pressure pain thresholds at both these tender points and control sites in healthy participants in concert with novel therapies will be important to increase the understanding of alterations in pain processing in these patients.

One method of therapy has been realized in the form of non-invasive cortical stimulation (NICS) with the primary motor cortex (M1) being the most effective target for the modulation of both experimental and chronic pain [3], [4]. Particular efficacy for NICS has been found in patients with musculoskeletal pain [5]–[8]. However a lack of understanding of the underlying physiological mechanisms has limited these techniques progress in clinical applications.

In this study we investigate the physiological mechanism of a novel NICS protocol in healthy adults. Pressure pain thresholds were used as an index of pain sensitivity so as to aid translation of our findings to a musculoskeletal pain population in future studies. There are currently very few studies assessing the efficacy of NICS on this clinically useful pain modality, which needs to be addressed [3].

### Non-invasive Cortical Stimulation (NICS)

There are two main methods of NICS; transcranial direct current stimulation (tDCS) and repetitive transcranial magnetic stimulation (rTMS) which are safe, easy to use and associated with relatively negligible side effects [3]. Both techniques can produce bidirectional after-effects on cortical excitability but depend on different stimulation parameters and physiological mechanisms [9]. tDCS has been hypothesized to modulate the resting membrane potentials of axons, altering the endogenous excitability of the target cortical area. It is primarily a modulatory technique with effects dependent on the polarity of stimulation used. In action, cathodal tDCS diminishes cortical excitability through the hyperpolarisation of neurons whereas anodal leads to neuronal depolarisation and increased cortical excitability [10]. However, the underlying mechanisms of tDCS require further research as recent studies have found that tDCS may not only modulate neurons but could also manipulate glial cells. Calculations indicated that transmembrane potential change is of similar magnitude as observed physiologically in astrocytes during neuronal activation [11]. In contrast rTMS is a stimulatory technique as it can generate propagated action potentials leading to the activation of neural circuits [3]. rTMS is frequency dependent with low frequencies (1 Hz or less) associated with decreased cortical excitability and high frequencies (20–50 Hz) associated with increased excitability. It is this mode of high frequency rTMS which has been established as a method of pain treatment with frequencies ≥10 Hz necessary to significantly modulate pain thresholds [3], [12]. Yet, high frequency rTMS can produce unpleasant scalp sensations which can be uncomfortable. Some patients in rTMS clinical trials have been unable to tolerate this sensation and have dropped out, which can hinder the use of rTMS as a form of pain management [13].

### Combined Paradigm (tDCS-rTMS)

It has been suggested that the initial state of the motor cortex may play a key role in the plastic effects of rTMS. Siebner *et al*., demonstrated how a combined paradigm using tDCS to “prime” or “precondition” motor cortex can successfully shape the conditioning effects of subsequent rTMS [14]. Results show that facilitatory preconditioning via anodal tDCS led to an overall inhibition of cortical excitability after subsequent stimulation by low frequency 1 Hz-rTMS and of particular note, inhibitory preconditioning via cathodal tDCS reverses the expected suppressive effects of low frequency 1 Hz-rTMS leading to an overall increase in cortical excitability. Thus, primed 1 Hz-rTMS can effectively simulate the result of high frequency rTMS on the human motor cortex. Similar studies suggest that this effect is mediated by homeostatic mechanisms whereby the human motor cortex stabilizes corticospinal excitability within a physiologically useful range [14]–[16]. tDCS-primed rTMS may also prove a more robust protocol as tDCS alone has recently been found to induce highly variable responses [17]. These discoveries prompted the use of a combined paradigm in our pain modulation research.

### Pain Modulation

Previous work revealed that tDCS-primed 1 Hz-rTMS can be applied to enhance the modulation of experimental thermal pain thresholds [18]. Inhibitory preconditioning via cathodal tDCS successfully produced increased thermal pain thresholds after subsequent stimulation of 1 Hz rTMS, simulating the physiological effects of high frequency rTMS whilst being a better tolerated method of intervention.

In our present study the technique of tDCS-primed 1 Hz rTMS is applied to pressure pain thresholds. Pressure pain has been proposed as a particularly useful modality, in that it is more directly related to a variety of clinical outcomes in a diverse range of pain patients [19], [20]. In the past, manual pressure pain assessment using traditional algometers has been documented to suffer from numerous limitations. This is predominantly due to human error of the operator as they were originally required to manually generate accurate and repeatable force profiles involving controlled ramp rate, consistent stimulus duration and inter-stimulus intervals. This experiment employs a computerized algometer (Medoc, Ramat Yishai, Israel) which acts to standardize the testing procedure and avoid operator-related error. This may allow pain researchers to attain accurate and repeatable force profiles which would be suitable for integration into a clinical setting, particularly in rheumatology patients.

Here, this preconditioning protocol is used to investigate the modulation of both cortical excitability and mechanical pressure pain which may prove useful in the translation of NICS effects between experimental and chronic pain groups.

## Materials and Methods

### Ethics Statement

The study was performed in accordance with the Declaration of Helsinki and approved by the Faculty of Health Sciences Research Ethics Committee, Trinity College Dublin. All participants provided written informed consent to participate in the study.

### Participants

Fifteen healthy male volunteers (mean age, 24.5 years ±3.4 SD, 12 right handed) were recruited to take part in the research study, by advertisement. Participants were approved to partake in the project once they completed a medical questionnaire which was deemed suitable by a qualified medical professional. Subjects with any previous or concomitant psychiatric or neurological disease, any conditions associated with acute or chronic pain or with somatosensory abnormalities were excluded. All participants were able to understand the QST protocol instructions and gave their written informed consent. All were completely naïve to the purpose of the experimental procedures.

### Quantitative Sensory Testing (QST)

**Figure 1:**
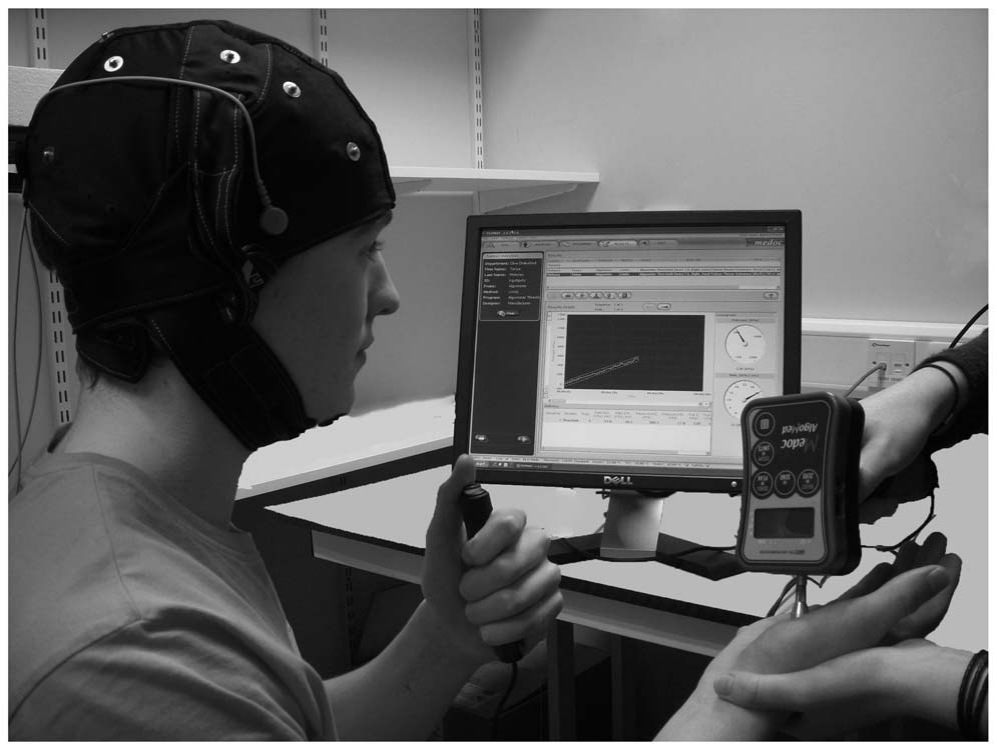
Pressure Pain Assessment Set-up. Handheld algometer used to determine pressure pain thresholds (PPT) of the right palmar thenar eminence via the Medoc Pathway System. The algometer was held perpendicular to the target site and applied according to computer controlled audio and visual prompts, with PPT detection reported via push-button response unit. tDCS was applied using the fitted MindCap with electrodes fixed in the C3/F8 position.

In order to determine pressure pain thresholds (PPT), a QST protocol was conducted using a standard digital algometer (FDIX; Wagner Instruments, Greenwich, CT) computer-controlled through The Medoc Pathway System (Medoc, Ramat Yishai, Israel). The algometer comprised of a pressure gauge and a 1 cm^2^ rubber plunger tip with a digital display of force in increments of 0.01 KPa. Real-time, visual and auditory feedback allowed the investigator to control and monitor applied pressure rates. The computerized algometer applied pressure stimuli according to the method of limits algorithm within the Pathway software and simultaneously recorded thresholds via a push-button response unit (Figure 1).

**Figure 2:**
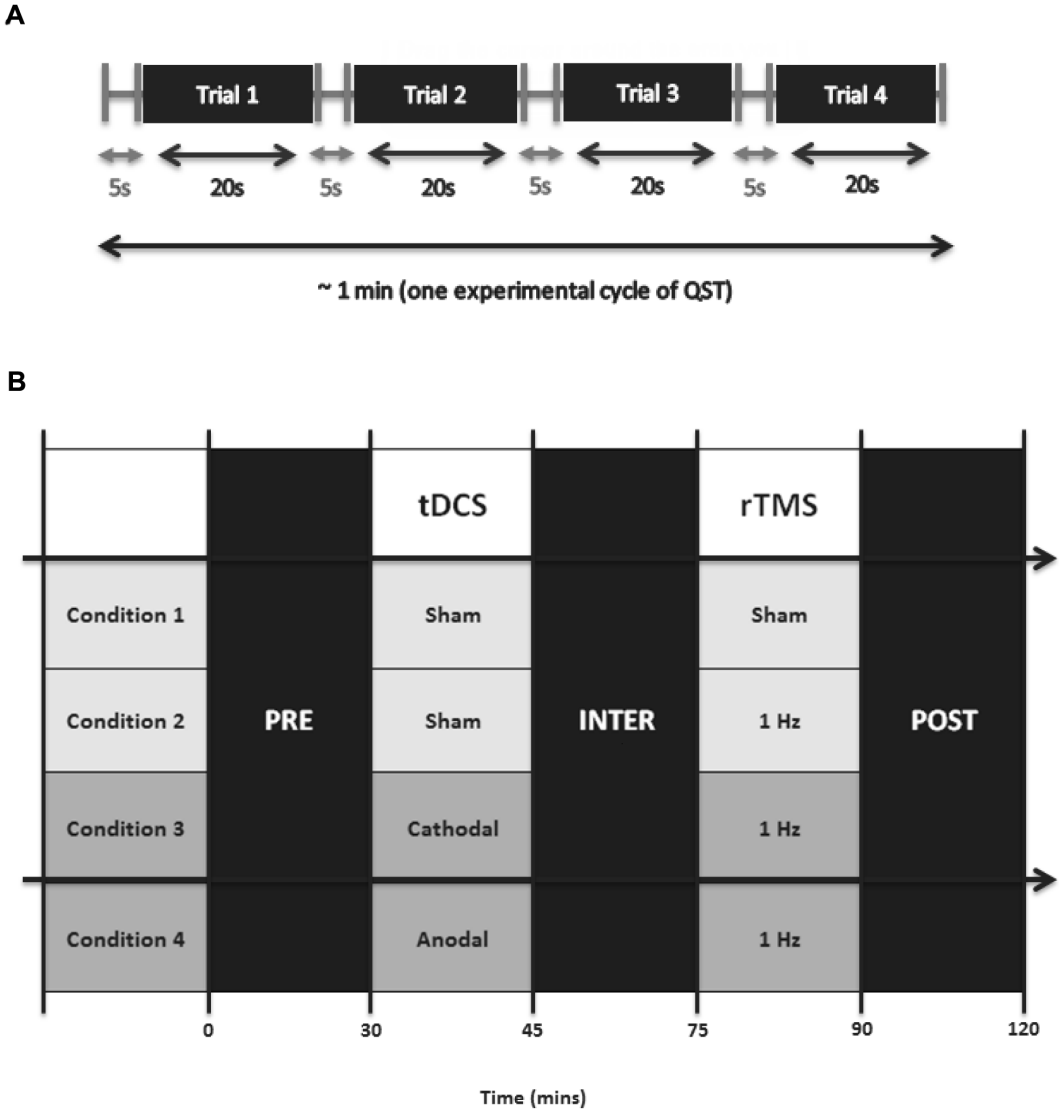
Experimental Approach. Panel A illustrates one cycle of pressure pain threshold (PPT) measurement using quantitative sensory testing (QST). Panel B depicts the timeline of the experimental sessions over the testing period. Experiments were conducted on the same day at the same time, every week, for experimental accuracy in data collection.

PPT was defined as the amount of force required to elicit a sensation of pain distinct from pressure or discomfort or in other terms; the point at which pressure transitioned to discomfort or pain [21]. In measuring PPTs, the algometer was applied perpendicularly to the skin and lowered at a rate of approximately 30 KPa/s until PPT was reached. The palmar thenar eminence of the hand was targeted for PPT measurement due to its ease of accessibility and proximity to the stimulated FDI. The palmar thenar eminence has also been used in clinical assessments in patients with musculoskeletal pain as a designated control site, distant from defined tender points [22]. The threshold was tested 4 times with an interval of ∼5 s between each successive trial, as illustrated in Figure 2A. Each trial lasted 20–25 s in duration, thus a full QST cycle required a solitary moment of focused, direct testing. QST was performed both prior to (PRE) and immediately after (POST) the combined neurostimulation intervention.

### Electromyograms and determination of stimulation parameters

Motor evoked potentials (MEPs) were recorded using surface electromyography (EMG) from the right first dorsal interosseous (FDI) muscle using conductive adhesive Ag/AgCl electrodes (Tyco Healthcare, Mansfield, UK) in a belly tendon montage. EMG signals were amplified, band-pass filtered (10–50 Hz) and sampled at 1,000 Hz using an Octal BioAmp (AD Instruments, Oxford, UK). The peak-to-peak amplitudes of the MEPs were then analysed off-line using custom-written software. The optimal placement for stimulation was defined as the site where single pulse TMS resulted in the largest MEP in a consistent fashion. These biphasic pulses were provided using a Magstim Rapid^2^ stimulator (Magstim Company Limited, Whitland, Wales, UK) connected to a figure of eight double coil (70 mm). The coil was placed tangentially to the scalp, with the handle pointing backward and laterally at a 45° angle to the sagittal plane, inducing a posteroanterior current in the brain [23] The resting motor threshold (RMT) is defined as the minimum TMS intensity which achieved peak-to-peak MEP amplitude of ≥50 μV in the resting FDI muscle, in 3 out of 5 stimulations. The measured RMT was used to set the output for subsequent rTMS protocols.

### Measurement of Motor System Excitability

To detect current-driven changes of excitability, motor-evoked potentials (MEPs) of the right FDI following stimulation of its motor-cortical representational field were recorded. A train of ∼20 TMS-evoked MEPs were recorded at 3.5 s intervals at an intensity of 120% of each individual's RMT. This train of MEPs was carried out prior to (PRE), in between (INTER) and immediately after stimulation (POST).

### Preconditioning the left M1 using tDCS

The application of tDCS was performed to the hand area of the left motor cortex; C3 according to the international 10–20 EEG system, through the use of a HDC kit (Magstim Company Limited, Whitland, Wales, UK). Direct currents were applied through a pair of saline soaked (0.89%NaCl) synthetic buckskin electrodes (25 cm^2^) with the active electrode placed over C3 and the reference electrode positioned contralaterally above the right orbit. These electrodes were held in place with use of the MindCap (Magstim Company Limited, Whitland, Wales, UK). Each of the four experimental conditions involved the continuous application of a current of 1 mA intensity for a duration of 10 minutes, as these parameters proved successful in priming the motor cortex in previous experiments [14]. Most subjects were aware of the current flow as an itching/burning sensation was reported upon stimulation with both polarities. For sham stimulation, placement of the electrodes was identical to both cathodal and anodal conditions. Here, current flow increased gradually over a 5 s interval reaching the designated 1 mA to mimic the initial itching sensation of real tDCS. The stimulation was then terminated after 10 s, so that a conditioning effect on cortical excitability would not be induced [24].

### Stimulation of left M1 with 1 Hz –rTMS

Using each participant's individual RMT, the TMS protocol was set to produce repetitive TMS (rTMS) at the designated 90% of a participant's individual RMT. This sub threshold intensity was chosen for two reasons: First, the intensity is likely to activate the corticospinal neurons trans-synaptically avoiding direct activation of these neurons [25]. Second, it was reasoned that sub threshold intensity may produce a relatively “weak” after-effect that would be more susceptible to the effects of pre-conditioning [14]. rTMS was then carried out at a frequency of 1 Hz with a train of 900 pulses, each of 1 s duration with a 1 s wait period (15 min total duration). Sham rTMS was administered with the coil tilted at a 45° angle from the surface of the head, discharging the same number of stimuli at the same rate as real rTMS. All subjects were naive to TMS, hence accepted the explanation that differences in scalp sensation between sham and real rTMS were due to stimulation at different intensity levels [26]. All rTMS protocols were carried out in accordance with the outlined safety guidelines [27].

### Experimental Protocol

A sham-controlled, single-blind study was conducted, consisting of four separate experiments, to determine how priming the motor cortex using tDCS may enhance the effects of rTMS on the modulation of pressure pain thresholds in healthy volunteers. The four conditions consisted of;

1. Sham tDCS – Sham rTMS
2. Sham tDCS – Real rTMS
3. Cathodal tDCS – Real rTMS
4. Anodal tDCS – Real rTMS

Each treatment was performed on the same day, at the same time over a period of 3 weeks in order to avoid any potential issues regarding a variation of pain thresholds due to circadian rhythms. Figure 2B illustrates a simple timeline of the treatment order and the time involved in each section. Overall, each experimental treatment takes approximately 2 hours in duration.

Each experiment was performed by the same, trained female examiner and at the same time of day. Sessions lasted approximately 2 hours for each condition, with participants kept conscious and alert by being addressed by the examiner during breaks from stimulation. An interval of one week was required between active testing conditions to avoid any interference that could be caused by the after-effects of stimulation. Treatment 1 and 2 acted as control sessions and were therefore suitable to be performed on the same day without risk of interference. Sham, cathodal and anodal tDCS-primed – 1 Hz rTMS interventions were presented in a pseudo randomized sequence and participants were unaware of the presented condition.

### Statistical Analysis

Collected data was first tested for normality of distribution using the Shapiro-Wilk's test within the SPSS statistical package (Version 20, IBM, New York, US). MEP data required transformation to square root in order to address moderate positive skew. All analyses were carried out using normally distributed data.

Test-retest reliability of PPT baseline measurements for each condition was calculated using SPSS based on a single rating, absolute agreement, 2-way mixed effect model (ICC_2, 1_). This statistic was used a measure of internal consistency and reproducibility across all conditions.

A two-way repeated measure (4×2) ANOVA was carried out to test the effects of Condition (Anodal, Cathodal, Control 1 and Control 2) and Time (Pre and Post) on the PPT data. A separate two-way repeated measure (4×3) ANOVA was used to assess the effects of Condition (Anodal, Cathodal, Control 1 and Control 2) and Time (Pre, Inter and Post) on MEP values. Planned contrasts were employed to assess statistical significance within each model. Values are presented as the mean of each participant's mean ± the standard error of the mean (SEM). A confidence level of 0.05 was set for all statistical tests.

## Results

Fifteen healthy male participants completed all protocols designed to determine if preconditioning the left M1 using tDCS (anodal, cathodal, sham) can modulate baseline cortical excitability and thereby shape the subsequent effects of low frequency rTMS (1 Hz) on pressure pain thresholds. None of the participants experienced any adverse side effects during or after the neurostimulation sessions.

### Quantitative Sensory Testing (QST) Analysis

Pressure pain threshold testing was found to be reliable on a test-retest basis across all conditions with an average baseline of 360±3.7 KPa, an intraclass correlation coefficient (ICC) value of 0.9 and a 95% confidence interval of 0.798–0.960. This indicates test-retest reliability to be highly consistent, allowing for greater statistical power in further analysis.

**Figure 3:**
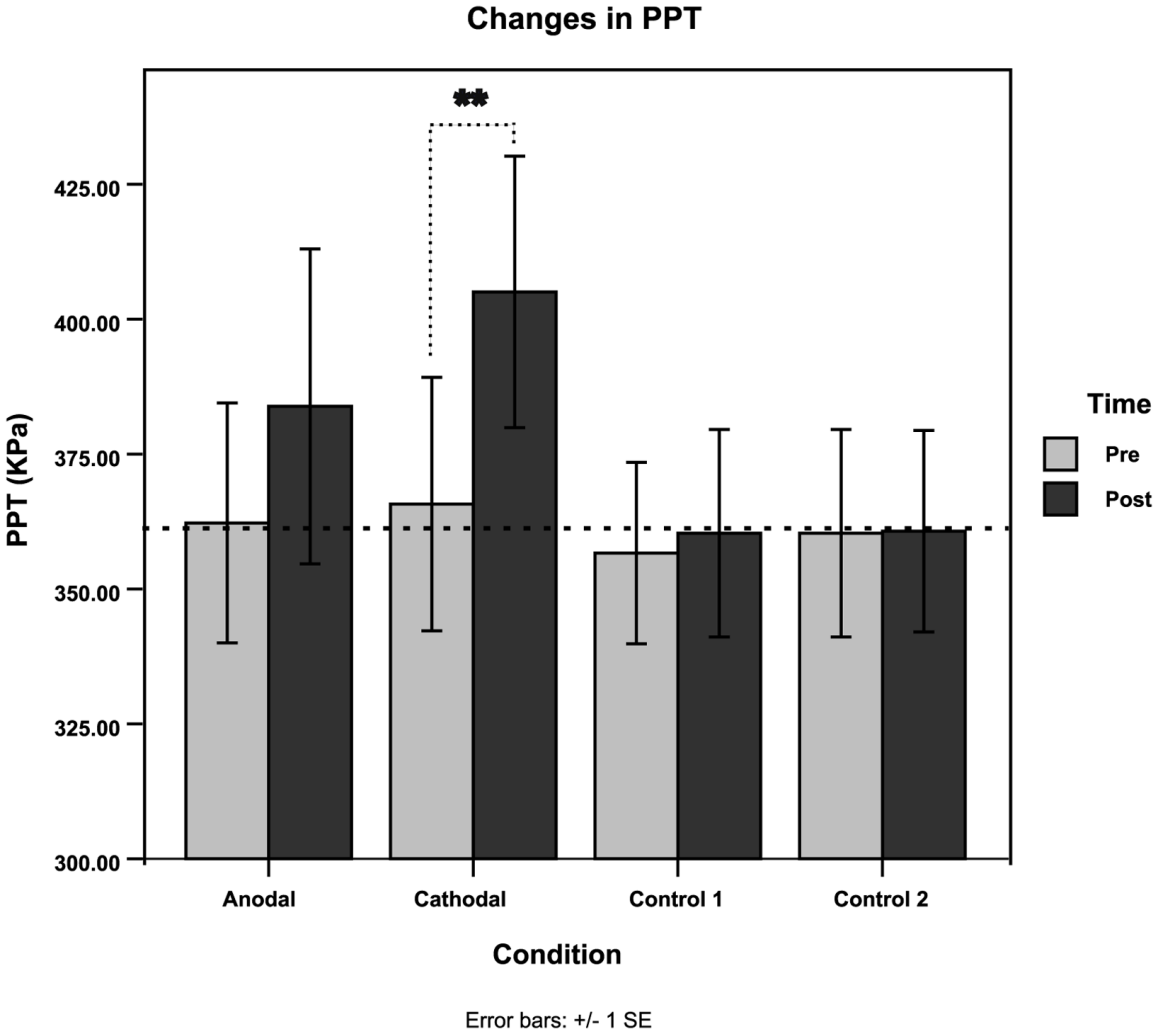
Changes in Pressure Pain Thresholds (PPT). Raw pressure pain thresholds (PPT) averaged and presented across all conditions, prior to stimulation (PRE) and immediately after stimulation (POST). Dotted line represents the average baseline of PPTs across all participants ∼361.25 KPa. (*Denotes significance, **p<0.01 within the 4x2 ANOVA as a main effect of the factor Condition)

Raw QST results revealed a main effect for both Condition (*F*(2, 28) = 3.42, *p*<0.05, *η^2^_p_* = 0.196) and Time (*F*(1,14) = 5.02, *p*<0.05, *η^2^_p_* = 0.264), however the respective interactions were not statistically significant (*F*(3,42) = 2.17, *p* = 0.105, *η^2^_p_* = 0.135). Planned contrasts demonstrated cathodal tDCS-primed 1 Hz-rTMS produced a significant increase in PPT from a baseline threshold of 365.73±90.92 KPa to 405.07±97.44 KPa post neurostimulation in regards to both the effects of Condition (*F*(1,14) = 10.68, *p*<0.01, *η^2^_p_* = 0.433) and Time (*F*(1,14 = 5.02, *p*<0.05, *η^2^_p_* = 0.264) (as seen in Figure 3). There was no statistical difference detected for any of the other conditions.

### Cortical Excitability (MEP) Analysis

**Figure 4:**
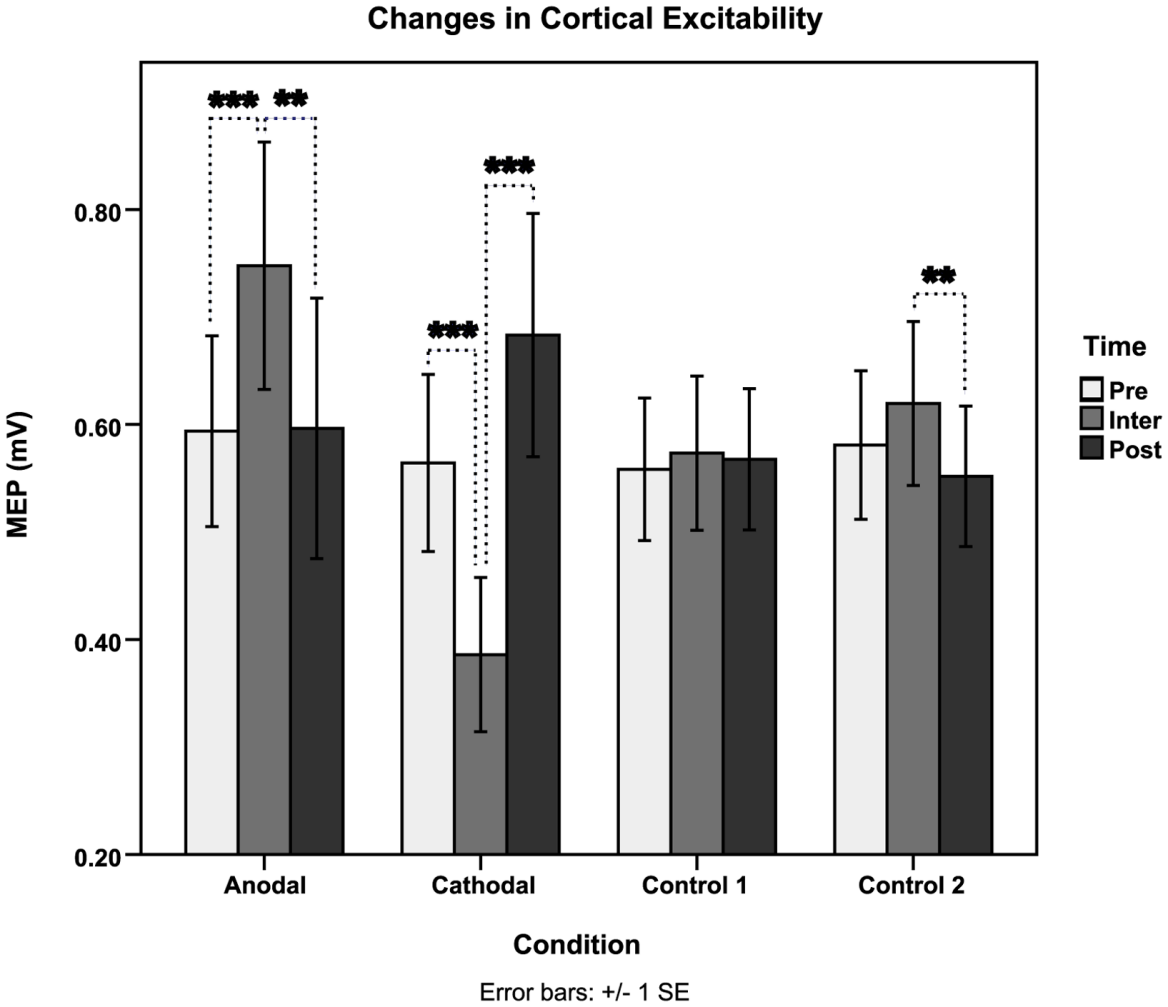
Changes in Cortical Excitability. Changes in cortical excitability were indexed using motor-evoked potentials (MEPs). Here MEP amplitudes are averaged and presented across all conditions, prior to stimulation (PRE), in-between stimulations (INTER) and immediately after stimulation (POST). (*Denotes significance. *(blue) infers significance PRE vs INTER, *(purple) INTER vs POST.

The ANOVA revealed a significant main effect for the interaction of the factors Condition x Time (*F*(2,29) = 12.06, *p*<0.001, *η^2^_p_* = 0.463) showing that this priming protocol did indeed have an effect on cortical excitability. However no significance was evident for the individual main effects of Condition (*F*(2,24) = 1.07, *p* = 0.35, *η^2^_p_* = 0.071) or Time (*F*(2,28) = 1.03, *p* = 0.371, *η^2^_p_* = 0.068).Within subject contrasts indicated a significant initial increase in MEP amplitude from a baseline of 0.6±0.34 mA (PRE) to 0.75±0.45 mA (INTER) after anodal tDCS priming (*F*(1,14) = 17.64, p<0.001, η2p = 0.558) and alternatively induced a significant decrease in amplitude ranging from 0.75±0.45 mA (INTER) to 0.6±0.47 mA (POST) due to the subsequent effects of 1 Hz rTMS (*F*(1,14) = 8.66, *p*<0.05, *η^2^_p_* = 0.382). Cathodal tDCS-primed 1 Hz-rTMS resulted in an opposite response of cortical activity as displayed in Figure 4. Cathodal tDCS priming produced an initial decrease in cortical excitability from a baseline of 0.56±0.32 mA (PRE) to 0.39±0.28 mA (INTER)(*F*(1,14) = 61.96, *p*<0.001, *η^2^_p_* = 0.816) and an equally significant increase in excitability from 0.39±0.28 mA (INTER) to 0.68±0.44 mA (POST) immediately after 1 Hz rTMS (*F*(1,14) = 25.93, *p*<0.001, *η^2^_p_* = 0.649). In comparing controls (Control 1: Sham tDCS-Sham rTMS vs Control 2: Sham tDCS-1 Hz rTMS) significant change was only seen INTER vs POST with a decrease in MEP size from 0.62±0.3 mA to 0.55±0.25 mA (*F*(1, 14) = 9.56, *p*<0.01, *η^2^_p_* = 0.406).

## Discussion

In this study we demonstrate that cathodal tDCS-primed 1 Hz-rTMS of the M1 can increase pain thresholds resulting in decreased sensitivity to experimentally provoked pressure pain. Further, cathodal tDCS-primed 1 Hz-rTMS was found to significantly increase cortical excitability, between the modified baseline levels (INTER) and final measurements (POST). Contrastingly, anodal tDCS-primed 1 Hz-rTMS produced a significant decrease in cortical excitability INTER vs POST with no effect on pain thresholds. These changes in excitability may provide a physiological basis for the observed pain modulatory effect.

### tDCS-primed 1 Hz-rTMS Alters Cortical Excitability Modulation

Prior to our exploration of homeostatic plasticity and pain threshold modulation, 1 Hz-rTMS had been found ineffective in pain relief and unworthy of further research in the field [28]. As a general rule, low frequency rTMS (<5 Hz) is thought to facilitate a depression in neuronal excitability whilst high frequency rTMS (>5 Hz) has been shown to increase neuronal excitability and consequently lead to a parallel increase in pain thresholds [26]. However, our investigation has found tDCS-primed 1 Hz-rTMS successful in enhancing the effects of low frequency rTMS and the modulation of pressure pain thresholds.

Our findings suggest that rTMS-induced plastic changes depend on the functional state of the motor cortex, which can be modified using tDCS, consistent with previous research [14], [15], [29]. These have been interpreted in the context of “homeostatic plasticity”. This concept of brain plasticity refers to the negative feedback mechanisms in place to regulate the activity of neural circuitry and prevent them from becoming hyper- or hypoactive [30]. The original model of homeostatic plasticity was formalized as the Bienenstock-Cooper-Munro (BCM) rule of bidirectional synaptic plasticity [31], where the threshold for long term potentiation(LTP)/depression(LTD) induction is not constant, but instead varies as a function of previous activity of the post synaptic neuron. Bienenstock, Cooper and Munro proposed the existence of a sliding modification threshold for synaptic plasticity, in that; the threshold for LTP induction increases if the previous level of post synaptic neuronal activity was high, but decreases if it was low, and vice versa for LTD induction where; the threshold for LTD induction decreases if post synaptic activity was high and increases if it was low.

Current preconditioning techniques seek to manipulate these homeostatic mechanisms. In effect, tDCS is used to augment background motor cortical excitability and thus enhance the cortical plastic changes induced by subsequent low-frequency rTMS. In the present study; we used the mean MEP amplitude as an index of the changes in primary motor cortex excitability. Of particular importance, participants displayed an increase in cortical activity in response to 1 Hz rTMS when previously inhibited by cathodal tDCS, successfully reversing the expected suppressive effects of 1 Hz rTMS. Contrastingly, the same 1 Hz rTMS protocol decreased the excitability of the motor cortex back to baseline levels after cortical excitability had been enhanced by a preceding anodal tDCS.

The level of homeostatic regulation appears to be dependent on the intensity of the stimulation applied. A study using tDCS to precondition the motor cortex before subsequent stimulation via high frequency 5 Hz-rTMS reported effects in agreement with our findings [15]. Healthy participants were found to demonstrate a decrease in cortical excitability in response to 5 Hz-rTMS when the motor cortex had been previously enhanced via facilitatory anodal tDCS priming. In effect, this may have resulted in a destabilizing potentiation of neuronal activity which required an increased level of homeostatic regulation leading to an ultimate depression of cortical excitability. In line with this theory, an equal level of homeostatic regulation would be appropriate in the case of our protocol comprised of cathodal tDCS-primed low frequency 1 Hz-rTMS, which would result in an overall extreme depotentiation of neuronal activity if it were not for the action of negative-feedback mechanisms eliciting an overall increase in cortical excitability.

Conversely, the same 5 Hz-rTMS protocol induced a rise in cortical excitability to return to baseline values after the motor cortex had been inhibited by a preceding session of cathodal tDCS [15]. Therefore combinations such as cathodal tDCS-primed 5 Hz-rTMS and our anodal tDCS-primed 1 Hz-rTMS did not provoke an equal level of homeostatic regulation and merely resulted in the return of MEP amplitudes to baseline. The nature of the biological mechanisms that generate this stability have yet to be elucidated, but in a recent paper Turrigiano et al, discusses how neuronal firing arises from the interplay between synaptic currents and the intrinsic firing properties of a neuron, providing two clear modes by which neurons could homeostatically regulate excitability [32].

### Pain Modulation

The present study reinforces the results of our previous work [18], in providing new evidence for the modulation of pressure pain thresholds using tDCS-primed 1 Hz-rTMS. However using the modality of pressure pain as an index and the same neurostimulation intervention, an increase in thresholds was seen post anodal and cathodal primed 1 Hz-rTMS, with significance present only for cathodal effects. This did not compare with the modulation of thermal thresholds as reported effects were significantly polarity dependent, demonstrating a decrease in thresholds post anodal tDCS-primed 1 Hz-rTMS and an increase post cathodal tDCS-primed 1 Hz-rTMS. While the physiological pathways underlying the perception of thermal stimuli are relatively understood within the scientific community [33], [34], those of mechanical stimuli such as pressure pain, have not yet been elucidated. Variations in the molecular mechanisms and related pathways between the perception and modulation of these two modalities may explain the contrasting effects seen within our studies.

How these forms of neurostimulation act on the M1 to modulate pain thresholds is also not yet fully understood. However many brain imaging studies have been carried out in relation to motor cortex stimulation (MCS), a technique comprised of an invasive, surgical procedure whereby electrodes are implanted onto the surface of the motor cortex. Results reveal activity changes in all neural structures involved in pain processing, such as the thalamus, anterior cingulate cortex (ACC), orbitofrontal cortex, insula, second somatosensory cortex (S2) or the periaqueductal grey matter (PAG) [35]–[38]. It has been hypothesized that part of the pain relief afforded by stimulation of the motor cortex could be through influencing thalamic activity, as the thalamus acts as the main relay centre for sensory information to the cortex. High frequency rTMS may directly activate the thalamus via corticothalamic projections and thereby suppress the transmission of sensory information via the spinothalamic pathway [39].Alternatively, it has been suggested that M1 stimulation may alter intracortical motor circuitry. According to Lefaucheur et al., high frequency rTMS (10 Hz) was found to re-establish intracortical inhibition in parallel with pain relief [40].

The molecular mechanisms underlying NICS-induced pain modulation also remain speculative. Lefaucheur et al., hypothesized that these analgesic effects may be mediated by the inhibitory GABAergic system [40]. This hypothesis has been supported by a recent study investigating an animal model of central pain due to spinal cord lesion [41]. It indicated that M1 stimulation produces its analgesic effects by enhancing GABAergic activity in the zona incerta, resulting in an increased inhibition of thalamic neurons involved in nociceptive processing.

We hypothesize that in altering the functional state of the M1 using cathodal tDCS before subsequent stimulation via 1 Hz rTMS, the resulting increase of pain thresholds mirror those of high frequency rTMS. This suggests the need for further research into the state dependent effects of rTMS in both healthy and patient populations with the aim of determining optimal protocols for clinical integration.

### Limitations

However the strength of our conclusions may be limited by our small, gender-specific sample size. Previous work in NICS and pain modulation have mainly used male participants due to stable hormone levels throughout testing periods with the aim of reducing inter-subject variability. A recent report outlined the presence of this strong male-orientated bias in experimental subject choice to date and issued a call for all experiments to be performed on both sexes [42]. Women are also greatly overrepresented among patients with chronic pain, with pain syndromes such as fibromyalgia occurring far more often in the female population [42]. Thus future studies would benefit from the extension of this work with the inclusion of female volunteers and a larger sample size to control for the increased variability. However, for initial insights into the physiological mechanisms underlying the efficacy of tDCS-primed 1 Hz rTMS, it is beneficial to reduce variability by selecting a healthy male cohort.

### Future in Clinical Practice

Future studies relating to clinical integration of these techniques must take into consideration the disease state of the individual. Pathophysiology is likely to alter the functional state of the cortex and lead to a different response pattern provoked by rTMS compared with healthy control subjects [43], [44]. Studies have demonstrated complete opposite results of this priming technique when performed on pain patients, suggesting a dysregulation in the inhibitory homeostatic mechanisms in various neurological disorders [45]. This knowledge can aid us in the progressive development of these preconditioning protocols seeking to amend these malfunctions in the neuronal circuitry and help to uncover novel, effective means of pain relief.

### Conclusion

Our study has found clear evidence to support the use of this tDCS-primed 1 Hz rTMS technique as a novel method in the modulation of pain. In line with current literature on metaplasticity, our results have shown cathodal tDCS-primed 1 Hz rTMS can increase cortical excitability and further, lead to a parallel increase of pressure pain thresholds, successfully producing a form of analgesia. These combined neurophysiological treatments have the potential to be beneficial in both experimental and clinical settings as a safe, non-invasive and innovative approach to pain modulation.
